# Detecting critical slowing down in high-dimensional epidemiological systems

**DOI:** 10.1371/journal.pcbi.1007679

**Published:** 2020-03-09

**Authors:** Tobias Brett, Marco Ajelli, Quan-Hui Liu, Mary G. Krauland, John J. Grefenstette, Willem G. van Panhuis, Alessandro Vespignani, John M. Drake, Pejman Rohani

**Affiliations:** 1 Odum School of Ecology, University of Georgia, Athens, Georgia, United States of America; 2 Center for the Ecology of Infectious Diseases, University of Georgia, Athens, Georgia, United States of America; 3 Laboratory for the Modeling of Biological and Socio-technical Systems, Northeastern University, Boston, Massachusetts, United States of America; 4 Bruno Kessler Foundation, Trento, Italy; 5 College of Computer Science, Sichuan University, Chengdu, China; 6 University of Pittsburgh, Department of Health Policy and Management, Pittsburgh, Pennsylvania, United States of America; 7 University of Pittsburgh, Department of Epidemiology, Pittsburgh, Pennsylvania, United States of America; 8 University of Pittsburgh, Department of Biomedical Informatics, Pittsburgh, Pennsylvania, United States of America; 9 ISI Foundation, Turin, Italy; 10 Department of Infectious Diseases, College of Veterinary Medicine, University of Georgia, Athens, Georgia, United States of America; Yale School of Public Health, UNITED STATES

## Abstract

Despite medical advances, the emergence and re-emergence of infectious diseases continue to pose a public health threat. Low-dimensional epidemiological models predict that epidemic transitions are preceded by the phenomenon of critical slowing down (CSD). This has raised the possibility of anticipating disease (re-)emergence using CSD-based early-warning signals (EWS), which are statistical moments estimated from time series data. For EWS to be useful at detecting future (re-)emergence, CSD needs to be a generic (model-independent) feature of epidemiological dynamics irrespective of system complexity. Currently, it is unclear whether the predictions of CSD—derived from simple, low-dimensional systems—pertain to real systems, which are high-dimensional. To assess the generality of CSD, we carried out a simulation study of a hierarchy of models, with increasing structural complexity and dimensionality, for a measles-like infectious disease. Our five models included: i) a nonseasonal homogeneous Susceptible-Exposed-Infectious-Recovered (SEIR) model, ii) a homogeneous SEIR model with seasonality in transmission, iii) an age-structured SEIR model, iv) a multiplex network-based model (Mplex) and v) an agent-based simulator (FRED). All models were parameterised to have a herd-immunity immunization threshold of around 90% coverage, and underwent a linear decrease in vaccine uptake, from 92% to 70% over 15 years. We found evidence of CSD prior to disease re-emergence in all models. We also evaluated the performance of seven EWS: the autocorrelation, coefficient of variation, index of dispersion, kurtosis, mean, skewness, variance. Performance was scored using the Area Under the ROC Curve (AUC) statistic. The best performing EWS were the mean and variance, with AUC > 0.75 one year before the estimated transition time. These two, along with the autocorrelation and index of dispersion, are promising candidate EWS for detecting disease emergence.

## Author summary

Emerging and re-emerging infectious diseases, such as Ebola and measles, present urgent public health challenges and threaten the progress made towards eliminating the global burden of disease. Consequently, a crucial activity in modern epidemiology is developing methods of anticipating (re-)emerging disease outbreaks. Early-warning signals (EWS) are a proposed method for detecting disease (re-)emergence, based on critical slowing down (CSD), a dynamical phenomenon present in systems approaching transition points. The presence of CSD preceding disease (re-)emergence has been comprehensively demonstrated in a range of low-dimensional epidemiological models. For EWS to be useful, however, CSD needs to be a generic feature of (re-)emerging disease transmission dynamics, rather than being limited to specific models. To assess the generality of CSD, we carried out a simulation study of a hierarchy of models of a re-emerging measles-like infectious disease. We found that CSD is present in the dynamics of all the models studied, supporting its generality. In addition, we studied seven candidate EWS, and found that four are strong candidates for use in monitoring systems to detect disease (re-)emergence.

## Introduction

Critical slowing down (CSD) is a dynamical feature of systems approaching phase transitions, and has been investigated both theoretically [1–7] and experimentally [8–14] across the natural sciences. As the transition is approached, the stability of the systems’ equilibrium weakens, causing an increasing persistence of perturbations away from the equilibrium (the eponymous “slowing down”) [4]. The ubiquity of CSD has led to suggestions that the phenomenon may be exploited to develop mechanism-independent methods of anticipating impending transitions [5]. This has spurred the examination of various summary statistics that can detect the presence of CSD in time series data and may serve as early-warning signals (EWS) [5–7, 9–14]. Anticipating the emergence of novel pathogens (such as H7N9 avian influenza virus [15]) and the re-emergence of historically controlled infectious diseases (such as measles [16]) is an urgent problem for global public health [17, 18], to which EWS are potentially well suited [6, 7].

**Fig 1:**
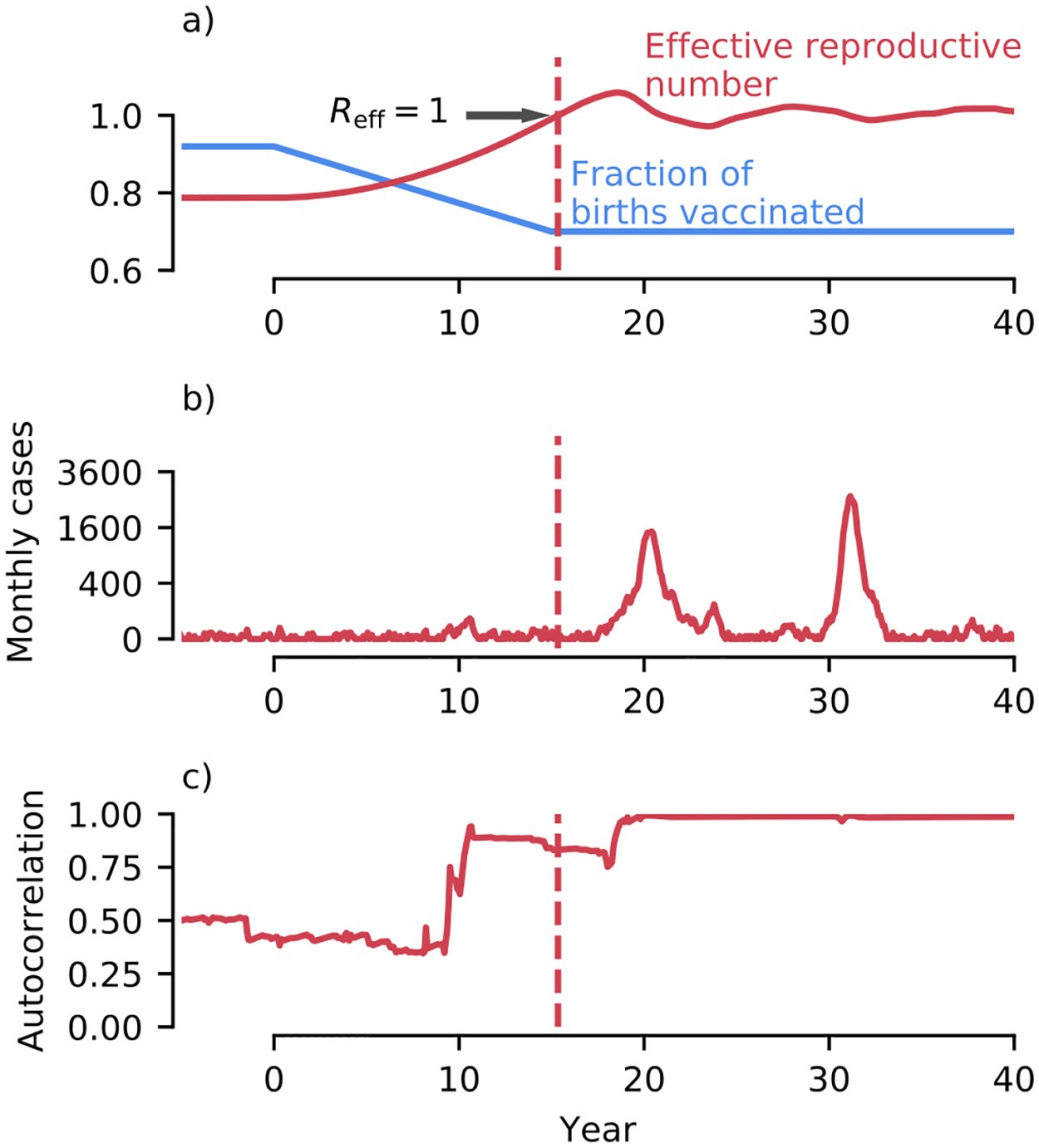
Example simulation of disease re-emergence using the nonseasonal SEIR model. Parameters were set to mimic transmission of a measles-like disease in a population of 10^6^ individuals, see Methods for model details and the full parameterization. a) The simulation was initialised above the herd immunity threshold, with 92% vaccine coverage. Starting in year 0, vaccine uptake of new born individuals drops linearly from 92% to 70% over 15 years. As vaccine uptake drops, *R*_eff_ increases, crossing the critical threshold *R*_eff_ = 1 shortly after 15 years. b) After the herd immunity threshold is crossed large outbreaks become possible, and endemicity is reestablished. c) Increases in early-warning signals (autocorrelation shown) precede the epidemic transition, enabling possible forewarning.

The key parameter that influences the threat posed by a (re-)emerging pathogen is the effective reproductive number, *R*_eff_, defined as the number of secondary cases a typical infectious individual causes [19]. *R*_eff_ can increase via multiple mechanisms, including changes in contact rates [20] and population immune profile [21, 22], environmental variation such as climate change [23], pathogen evolution (leading to evasion of immunity [24, 25] and host adaptation [26]), and declining vaccine uptake [16]. As *R*_eff_ increases the transmission dynamics undergo a phase transition (Fig 1a). Below the epidemic threshold, *R*_eff_ = 1, there is limited secondary transmission of the disease, however above the threshold large-scale epidemics and endemicity become possible (Fig 1b). The existence of CSD as *R*_eff_ approaches 1 has been comprehensively demonstrated in a range in low-dimensional epidemiological models (see for instance Fig 1c), including those with: seasonality in transmission [27], imperfectly reported data [28, 29], declining vaccine uptake [6] and vector-borne transmission [30]. One gap where the presence of CSD has not been demonstrated is in high-dimensional epidemiological models. For the purposes of this paper, we define a high-dimensional model as one possessing a large number of state variables (this is in contrast to dynamical definitions of dimensionality, which may be lower due to a separation of dynamical time-scales [31] or weak coupling between state variables [32]). By sacrificing analytical tractability, high-dimensional models are designed to provide a more realistic representation of the actual transmission dynamics of disease in nature [33–36] and thus serve as a bridge between low-dimensional models and the real world.

**Fig 2:**
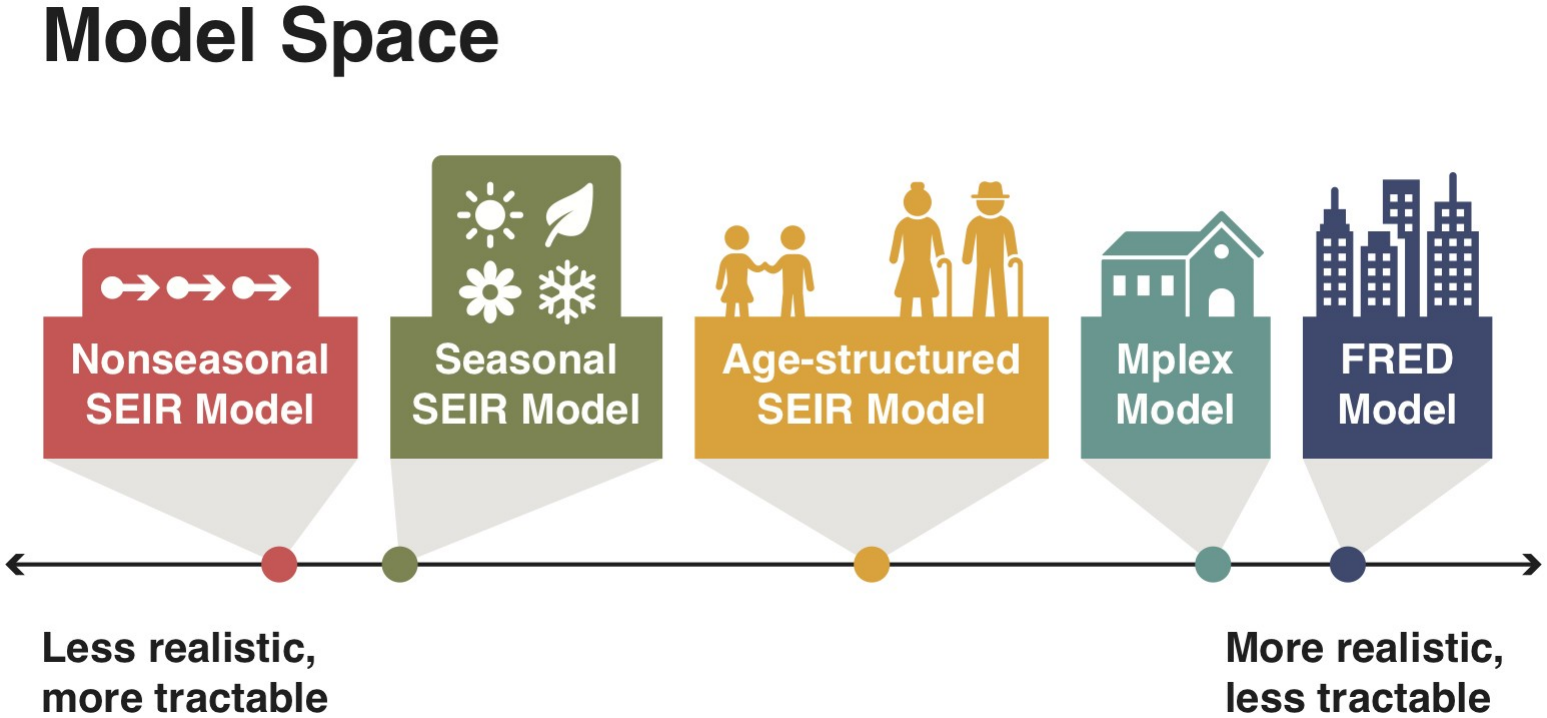
Representation of the trade off between tractability and realism in model construction. Models are positioned along the axis based on the relative complexity of the model, as determined by the number of state variables (the dimensionality) and model structure (the interactions between state variables). The nonseasonal SEIR model is the simplest model, with the FRED and Mplex models being the most complex. Simpler models lend themselves to mathematical analysis, while sacrificing realism. More complex models better represent reality, at the expense of analytical tractability.

The aims of this paper are to i) ascertain whether CSD is present in high-dimensional epidemiological models and ii) evaluate the performance of a range of EWS at detecting (re-)emergence. We studied five different transmission models, of varying dimensionality and structure (Fig 2). Three models were variants of the Susceptible-Exposed-Infectious-Recovered (SEIR) model, a canonical model of mathematical epidemiology: the basic nonseasonal SEIR model, the SEIR model with seasonality, and an age-structured SEIR model which has assortative mixing between age groups. In addition we considered i) a multiplex contact network model parameterised using socio-demographic data (referred to in this paper as the Mplex model) [37] and ii) FRED (A Framework for Reconstructing Epidemiological Dynamics), an agent-based modeling system [35]. We simulated a comparable re-emergence scenario with each model and, from the resulting time series, calculated seven candidate EWS (the autocorrelation, coefficient of variation, index of dispersion, kurtosis, mean, skewness and variance) previously proposed in the literature [28]. To assess whether the epidemic transition was preceded by CSD and detectable EWS, we first estimated the time of emergence (when *R*_eff_ = 1) for each model by fitting a Poisson transmission model using Bayesian MCMC. The presence of CSD prior to re-emergence was then established by inspecting the autocorrelation at lag 1 month. We assessed the operational performance of EWS, finding that four out of seven EWS (the autocorrelation, index of dispersion, mean and variance) are credible candidates for detecting disease re-emergence.

## Results

### Simulated time series

**Fig 3:**
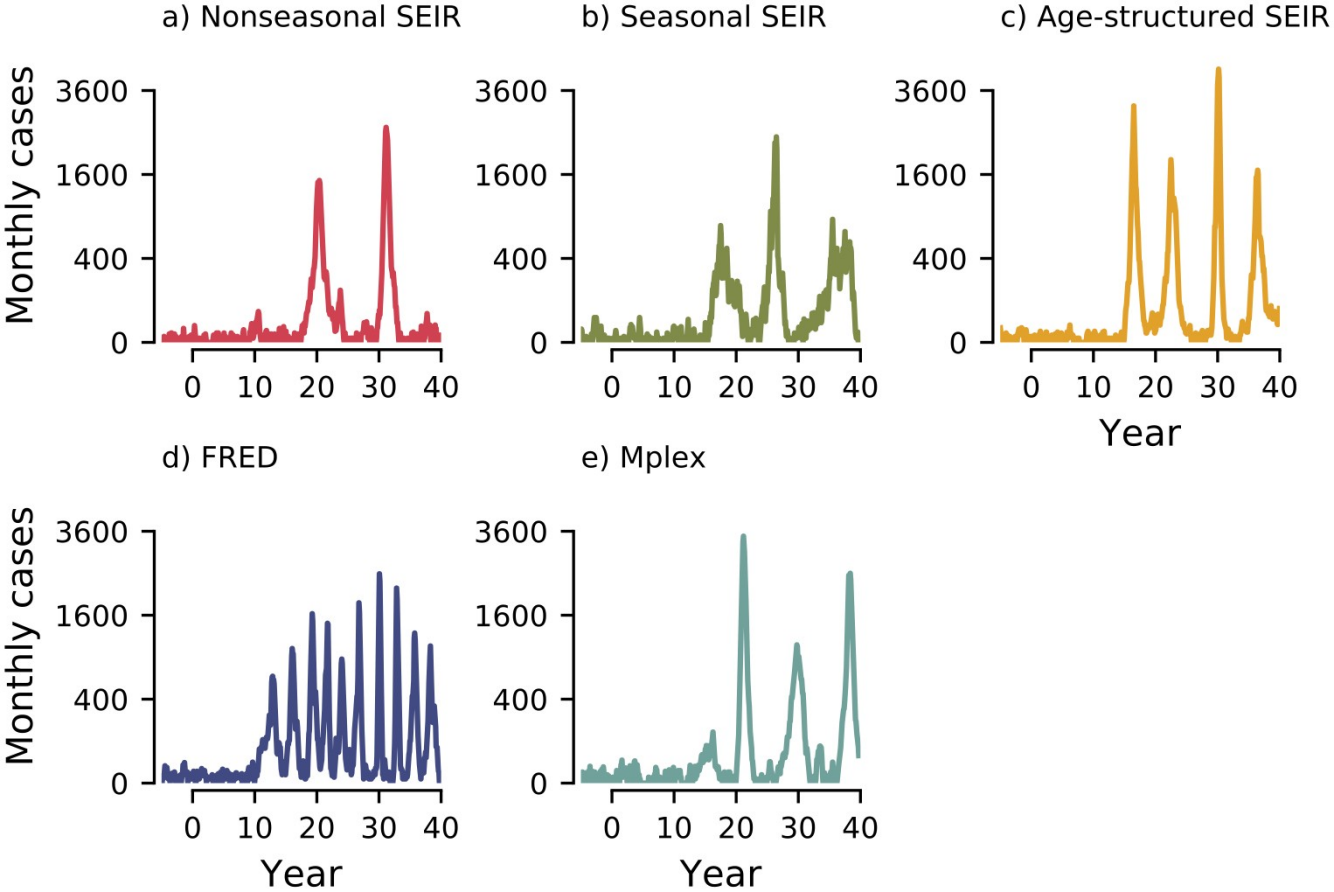
Example simulated time series of monthly cases for the five models (panels a–e). Each model was parameterised to have a herd immunity threshold around 90% vaccine coverage, and experienced the same decrease in vaccine coverage over the same time span as Fig 1a. Qualitatively, we see that the effect of declining vaccine coverage is model-structure dependent. For the time series shown, the time to the first major outbreak varies between 10 years for FRED (panel d) to 18 years for the nonseasonal SEIR model (panel a).

Representative simulated time series of monthly cases using each model are shown in Fig 3 (for experiment design and model details see Methods). During the herd immunity era (vaccine coverage at 95%, *t* < 0 years), monthly incidence was low in all models, with averages ranging from 1.42 cases for the age-structured SEIR model to 3.74 cases for FRED.

As vaccine coverage dropped (via a linear decrease in vaccine uptake from 95% to 70% over 15 years), incidence gradually rose until herd immunity was lost, and there was a transition to large outbreaks. We refer to the time of this transition as the time of emergence. Both the time of emergence and the outbreak dynamics after the transition varied among models. The nonseasonal and seasonal SEIR model both had long multi-year outbreaks, whereas all other models had more intense, short-lived epidemics.

### Time of emergence

**Fig 4:**
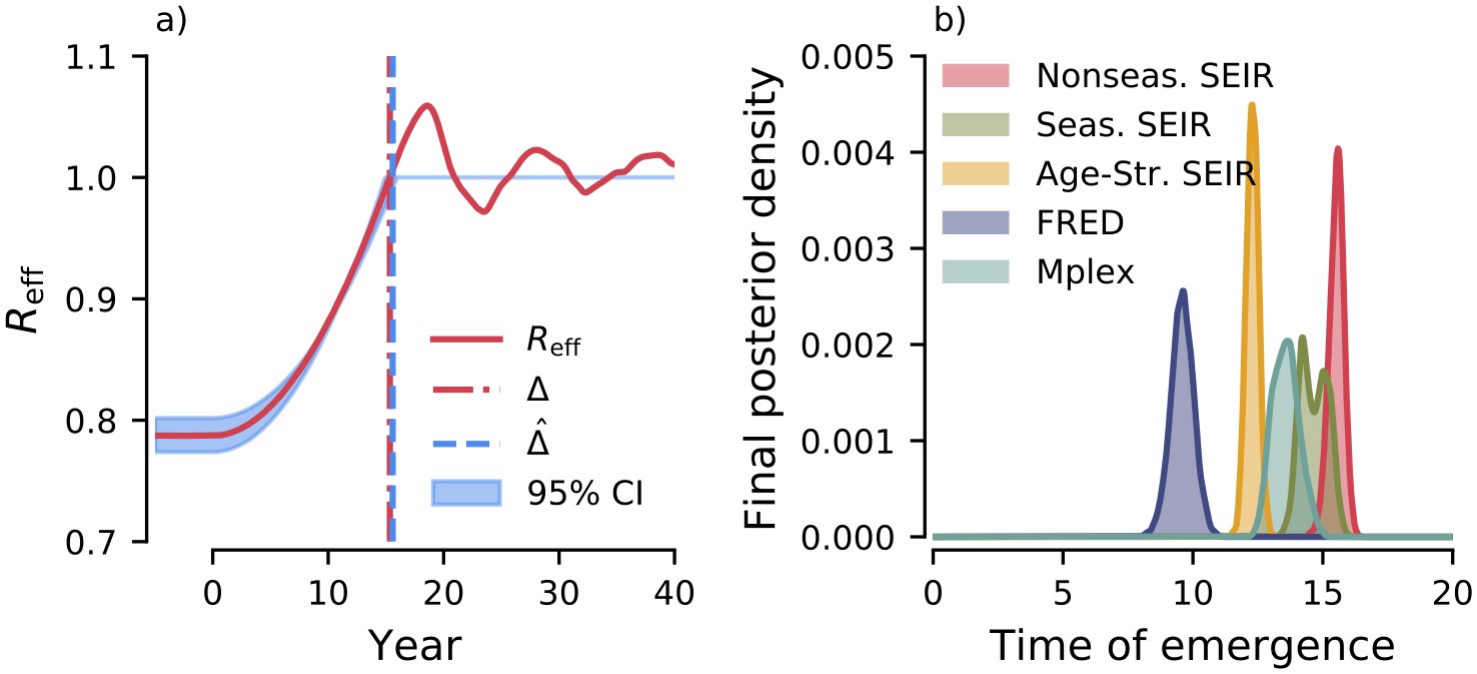
Estimating of time of emergence from case reports data. a) The Poisson transmission model assumes *R*_eff_ is a piecewise linear function of time, with a quadratic increase from $R_{\text{eff}}=R_{\text{eff}}^{i}$ at *t* = 0 to *R*_eff_ = 1 at *t* = Δ. The time of emergence, Δ, is estimated from the simulated data using Bayesian MCMC (see Methods). b) Final posterior density of the time of emergence. The MAP values of $\hat{\Delta }$ for each model are listed in Table 1.

In Fig 4a we show the effective reproductive number, *R*_eff_(*t*), and time of emergence, Δ, for the nonseasonal SEIR model. After fitting the Poisson transmission model to all 100 time series (see Methods), the *maximum a posteriori* (MAP) for the time of emergence is $\hat{\Delta }=15.59$ years after vaccination started decreasing. The posterior density for Δ is sharply peaked, with a 95% credible interval (CI) of [14.92, 15.95]. The MAP lies within 4 months of the true time of emergence, Δ = 15.3 years.

The remaining models have no analytical solution for Δ; the posterior densities $\pi^{M}\left(\Delta |{\left\{C_{j}\right\}}_{j=1}^{M}\right)$ after fitting the Poisson transmission model are shown in Fig 4b. The MAP estimates of $\hat{\Delta }$ and $R_{\text{eff}}^{i}$ are summarised in Table 1. Including seasonality in the SEIR model results in a bimodal posterior density (Fig 4b) with the MAP ($\hat{\Delta }=14.20$ years) roughly 1 year before that of the nonseasonal SEIR model. For the age-structured SEIR model, $\hat{\Delta }=12.28$ years. The posterior density is more sharply peaked around the MAP. The agent-based simulator FRED has the earliest time of emergence, $\hat{\Delta }=9.61$ years, whereas the Mplex model has an intermediary time of emergence, $\hat{\Delta }=13.63$ years. The posterior densities for both models are less sharply peaked than the age-structured SEIR model.

**Table 1:** Estimates of the time to emergence (Δ; in years) and initial reproductive number ($R_{\text{eff}}^{i}$) for each model (MAP point estimate and 95% credible interval).

Model	Δ		$R_{\text{eff}}^{i}$	
	MAP	95% CI	MAP	95% CI
Nonseasonal SEIR	15.59	[14.92, 15.95]	0.79	[0.77, 0.80]
Seasonal SEIR	14.20	[13.78, 15.47]	0.78	[0.76, 0.79]
Age-structured SEIR	12.28	[11.83, 12.67]	0.75	[0.73, 0.76]
FRED	9.61	[8.68, 10.30]	0.89	[0.88, 0.90]
Mplex	13.63	[12.67, 14.44]	0.81	[0.79, 0.82]

### Detection of critical slowing down

**Fig 5:**
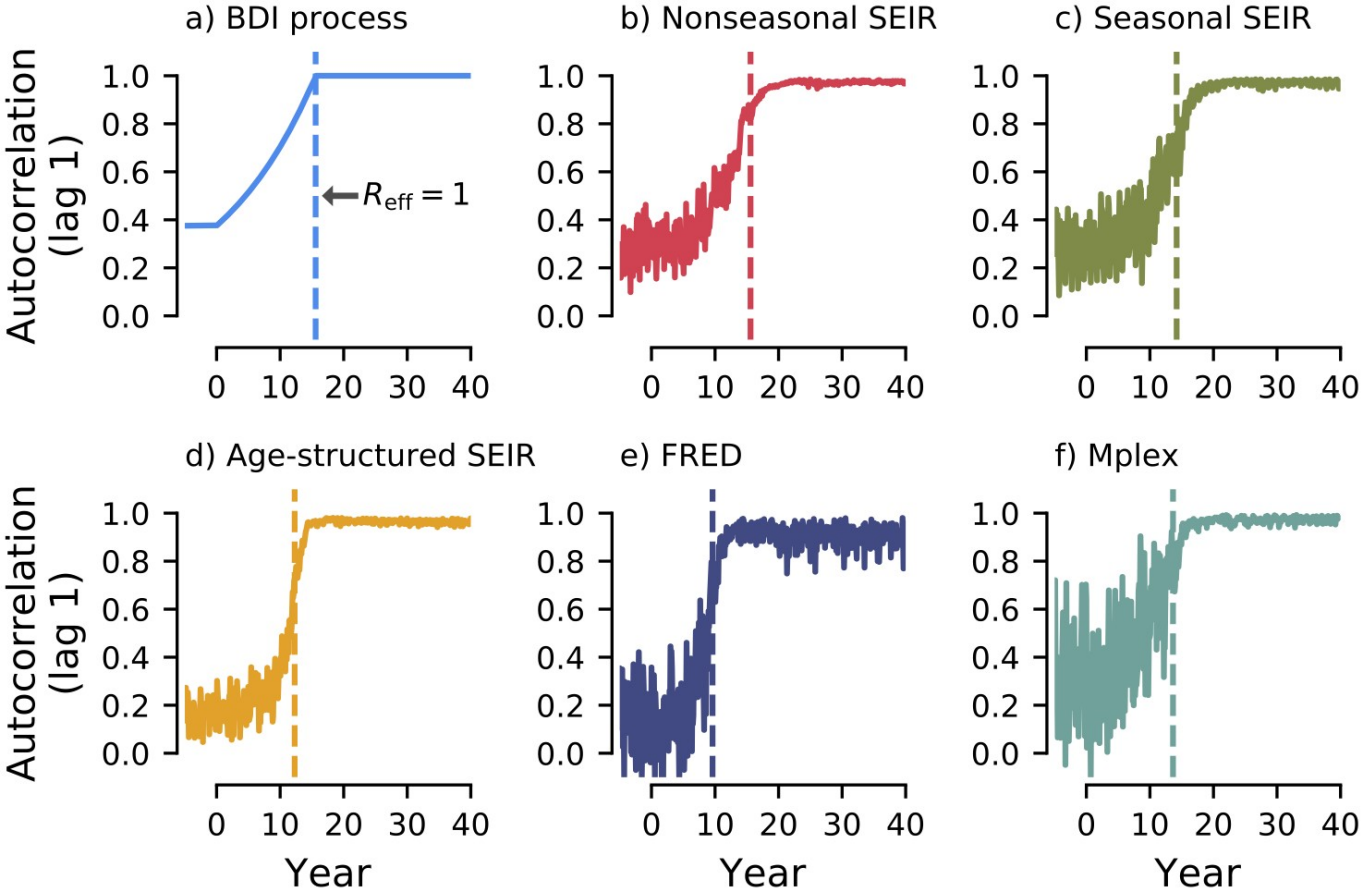
The autocorrelation at lag one month through time. a) Theoretical benchmark using the BDI process, given by Eq 11. b-f) Estimates for the autocorrelation calculated for each month from the ensemble of realisations. MAP estimates of the time of emergence, $\hat{\Delta }$, are indicated by dashed vertical lines. For all models, the autocorrelation increases as the time of emergence is approached, indicative of CSD.

As a theoretical benchmark, the autocorrelation of the Birth-Death-Immigration (BDI) process (see Methods) using a parameterization matched to the simulated SEIR model is shown in Fig 5a. As *R*_eff_ → 1, the autocorrelation increases and approaches 1, indicative of CSD.

For the five models studied in this paper (Fig 5b–5f) we also saw an increasing trend in the autocorrelation for $0<t<\hat{\Delta }$. Unlike for the BDI process, the autocorrelation did not reach 1 at the transition in any of these models, due to the effects of susceptible depletion and the speed of emergence [7]. Models with a faster speed of emergence (such as FRED, Fig 5e) had a lower autocorrelation at the time of emergence. The observed increase in autocorrelation for all models studied supports the hypothesis that CSD is a generic feature of epidemiological dynamics approaching the epidemic transition.

### Performance at detecting disease emergence

**Fig 6:**
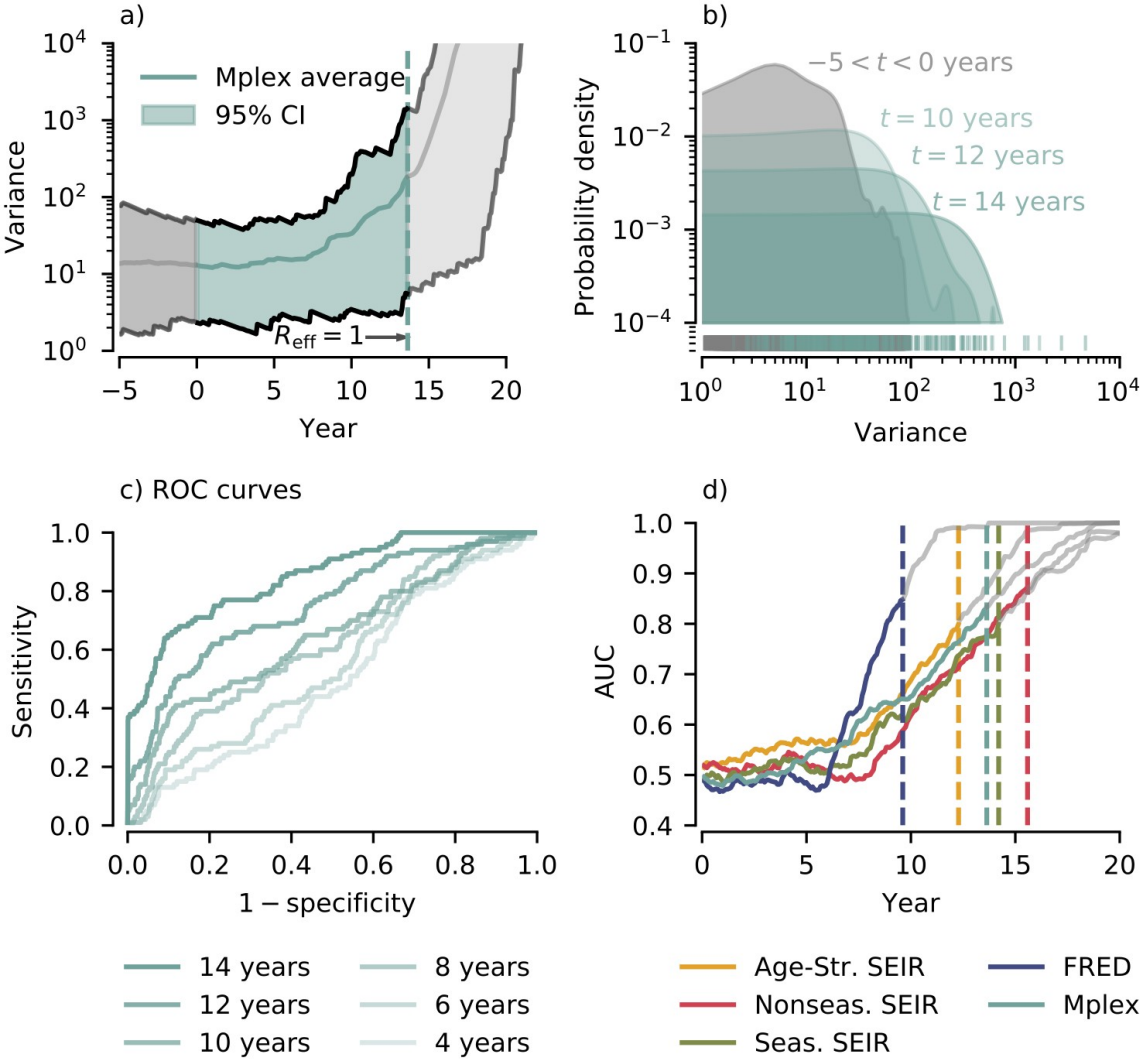
Performance of the variance at detecting emergence. a) Variance for the Mplex model calculated using an exponentially weighted moving window with a half life of 3 years. Mean and 95% credible interval calculated using 100 realizations. b) Test (green) and null (grey) probability densities for the variance. Probability densities found using kernel density estimation (see Methods). Null probability density calculated using all data points in the interval −5 < *t* < 0 years. Test probability densities shown for *t* = 10, 12, 14 years. c) ROC curves for the variance for the Mplex model shown for 2 year intervals. d) Area Under the ROC Curve (AUC) through time for the variance for each model. Vertical lines indicate the time of emergence.

Fig 6a shows the variance calculated using an exponentially weighted moving average for the Mplex model. Probability densities for the variance during the period −5 < *t* < 0 years (null period) and at the time points *t* = 10, 12, 14 years are shown in Fig 6b. As coverage dropped, the average over 100 realizations and the 95% confidence interval both shifted to higher values and the overlap of the null (−5 < *t* < 0 years) and test distributions decreased.

The decrease in distribution overlap is reflected in the Receiver Operator Characteristics (ROC) curve (for details see Methods). As *t* increased, the ROC curve moved towards the top left corner (Fig 6c) implying emergence became easier to detect using the variance. For all models the Area Under the ROC Curve (AUC) rose from 0.5 (uninformative classifier) after vaccine uptake started declining (Fig 6d). The AUC through time for the remaining EWS are presented in S1 Fig.

**Fig 7:**
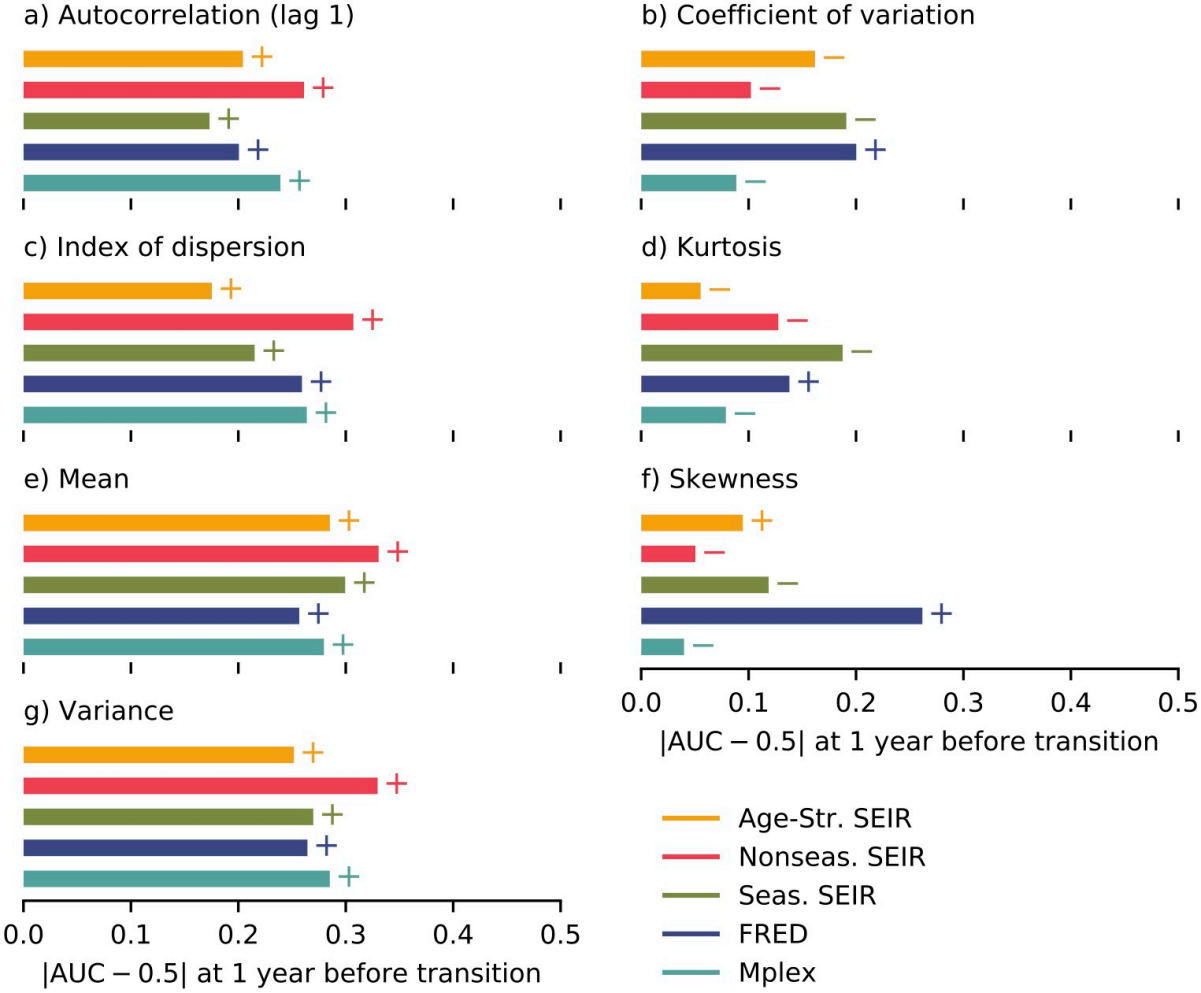
Summary of the AUC values one year before the transition. a–g) AUC values for each model for the EWS indicated in the panel. The + (−) symbols next to each bar indicate that the AUC is greater (less) than 0.5.

Performance at detecting emergence depended on both the EWS and the model. AUC values one year before the estimated time of emergence are summarised for each combination of EWS and model in Fig 7.

Most EWS consistently increased before the transition (indicated by a “+” in Fig 7). The exceptions were the coefficient of variation, kurtosis and skewness. For the coefficient of variation and kurtosis, one model (FRED) had AUC > 0.5 one year before the transition, whereas the remaining four models had AUC < 0.5. For the skewness, two models (FRED and the age-structured SEIR model) had AUC > 0.5. The inconsistency in the trends of these three EWS prior to the epidemic transition make them poor indicators of emergence.

For the other EWS (the mean, variance, index of dispersion and autocorrelation), performance was generally similar for each model. Using any of these EWS, emergence was easiest to detect in the nonseasonal SEIR model (which had the latest time of emergence), however there was no consistent order for the remaining models. Performance was generally slightly higher for the mean and variance (with AUC values ranging from 0.75 to 0.83) than for the autocorrelation and index of dispersion (AUC ranging from 0.67 to 0.81).

Our quantification of EWS performance is sensitive to i) the estimated time of emergence and ii) the lead time before the transition (chosen to be 1 year in Fig 7). Sensitivity to both these factors can be inferred from S1 Fig. For the four reliable EWS, the AUC rises with time after year 0 (when vaccine uptake started decreasing), as expected. The faster the change in AUC, the greater the sensitivity to both the estimate of the time of emergence and the lead time relative to the time of emergence. FRED, which has the earliest time of emergence, has the fastest rate of increase in AUC. For the remaining models, the rate of increase in AUC is comparable.

## Discussion

Research into critical slowing down and EWS preceding emerging disease outbreaks has, up to this point, focused on low-dimensional models that can be studied analytically [6, 7]. In formulating these models, a large number of simplifying assumptions are made, leaving open the question of whether CSD and EWS are unique to simple models, or are a more generic feature of epidemiological dynamics. In this paper, we addressed this question by studying five models with very different structures: two well-mixed models (the seasonal and nonseasonal SEIR model), an age-structured SEIR model with age-dependent contact rates, the Mplex model which explicitly modelled a contact network of schools and households, and FRED which simulates a synthetic population of interacting agents. We used each of these models to simulate the transmission dynamics of a measles-like vaccine preventable disease that was re-emerging due to declining vaccine uptake.

The first aim of this paper was to ascertain whether CSD was present in high-dimensional epidemiological models prior to the epidemic transition *R*_eff_ = 1. We detected CSD in all models before the critical transition. The observed ubiquity of CSD suggests it is intrinsic to re-emerging disease dynamics. In simple terms, we expect this is due to all of our models (and also infectious disease transmission in nature) sharing a common causal relationship: as vaccination coverage drops towards the herd-immunity threshold, the probability of longer chains of transmission increases. As explained in a previous study [7], this forms the dynamical basis for CSD in low-dimensional epidemiological models. Our study demonstrates that the additional dynamical complexities introduced in high-dimensional models do not serve to mask [38] or negate [39] the existence of CSD. Model structure did, however, have an impact on the time of emergence (the lag between vaccine coverage starting to decline and the effective reproductive number *R*_eff_ reaching unity).

The second aim was to evaluate the performance of a range of EWS, which we quantified using the AUC statistic. For most EWS (the autocorrelation, index of dispersion, mean and variance), performance increased as the time of emergence was approached. Performance of the other three EWS (the skewness, kurtosis and coefficient of variation) did not have a consistent relationship with time; whether the AUC for these three EWS increased or decreased prior to the transition was found to be model-dependent. These findings corroborate those of a previous study into the detectability of emergence using imperfect data [28], confirming that these three are, in isolation, unreliable EWS. Overall, the best performing EWS were the mean and variance, with AUC > 0.75 one year before the transition for all models. These two, along with the autocorrelation and index of dispersion, are promising candidate EWS for detecting disease emergence.

We focused in this study on the impact of dimensionality and model structure on the detectability of re-emergence, considering models in which the interactions between individuals were clustered in various ways (e.g. by age, school, neighborhood). However, to simplify the comparison, we did not consider social clustering of vaccine status—i.e. in the models studied, all new born individuals had an identical probability of receiving the vaccine. One factor that has been clearly implicated in recent measles outbreaks in high-vaccination countries is that unvaccinated individuals tend to be socially clustered [40]. As vaccine uptake declines, these clusters will change in size and composition, which can lead to different re-emergent dynamics [41]. Investigating whether CSD is present in settings with heterogeneous vaccine uptake, and what the impacts are for potential early-warning, is an pressing topic for further study.

There are many mechanisms that can drive disease (re-)emergence. Here, we have concentrated only on declining vaccine uptake, however CSD is theoretically predicted to be independent of the mechanism causing *R*_eff_ to increase. One particularly challenging mechanism of emergence that warrants further study are changes in the population structure itself [42]. For instance, rapid increases in population density and connectivity in West and Central Africa have been suggested to enhance the risk of emerging disease outbreaks such as Ebola [43]. High-dimensional models may play a key role in understanding these changing risks and EWS in monitoring them.

Our findings confirm that CSD is present in high-dimensional models, bridging a key gap between previous theoretical results for low-dimensional systems and the real world. Our results add further support to the hypothesis that CSD is a generic feature of (re-)emerging epidemiological dynamics driven by increases in *R*_eff_, and that the epidemic transition is preceded by detectable EWS. Developing detection methods that operationalise EWS and can inform public health bodies presents a clear future step.

## Methods

### Experiment design

To investigate the generality of CSD, we studied five transmission models with very varied structures undergoing the same epidemic transition: the loss of herd immunity in a population due to declining vaccine uptake. To provide a meaningful comparison, where possible all five models were assigned identical epidemiological and demographic parameters.

For all models, infection followed an SEIR-type sequence: upon infection susceptible (S) individuals enter a latent non-infectious stage (E), followed by an infectious stage (I), followed by eventual recovery (R). The mean latent period and infectious period are set to values appropriate for measles, 1/*ρ* = 8 days and 1/*γ* = 5 days, respectively [44]. We assume that infection is non-virulent (i.e. all individuals recover) and confers perfect life-long immunity. In each of the models, presence of the pathogen in the population was maintained by individuals contracting the infection from external sources (referred to as importation). In a fully susceptible population, on average one importation occurred per week, *ζ* = 1 week^−1^. The *per capita* rate of importation is given by the ratio *ζ*/*N*_0_ where *N*_0_ is the population size, and was uniform for all susceptible individuals. All models output weekly cases reports over the interval *t* = −10 to *t* = 40 years. We assumed perfect reporting (i.e. case reports equal the true number of weekly cases).

All models bar FRED had a mean population size *N*_0_ = 10^6^, a *per capita* annual birth rate of 0.013 and mean life expectancy of 75 years. The values for FRED were similar, matching those of Allegheny county, PA, USA (see below), specifically: *N*_0_ = 1.2 × 10^6^, a *per capita* annual birth rate of 0.011 and mean life expectancy of about 78 years.

The primary difference between the models was in the structure of the populations, i.e. in the dynamics of contacts between individuals. It is these contacts that facilitate disease transmission from infectious to susceptible individuals, with a probability given by the pathogens transmissibility. The details of the contact structures for each model are described in the following sections. While the contact structure varied widely between models, the basic reproductive number *R*_0_ (the average number of secondary cases an infectious individual causes in a fully susceptible population) was set to be roughly the same for all models to ensure comparability of results.

The models were driven through the epidemic transition via the same decline in vaccine uptake. The probability that a new born individual receives the vaccine *v*(*t*) decreased linearly from 0.92 to 0.70 over 15 years, starting in year 0. We assumed immunised individuals receive a perfect vaccine (i.e. with no primary vaccine failure, leakiness or waning of immunity [45]) at birth. By tuning the pathogen’s transmissibility, we fixed the herd immunity immunization threshold of each model to be around 90% coverage (in line with *R*_0_ ≈ 10). All models were therefore initialised above the herd immunity threshold. The timing of the epidemic transition depended on the details of the model structure (see below).

Our models all incorporated the effects of demographic stochasticity [46], hence we examined 100 realizations for each.

#### Nonseasonal SEIR model

The first model considered was the nonseasonal SEIR model with birth and death. The model included the effects of demographic stochasticity, modeling the transmission dynamics as a discrete sequence of jumps between states [44, 46]. Simulations were performed using the Next-Reaction Method (NRM) algorithm [47]. Unvaccinated individuals were born with rate {1 − *v*(*t*)}*αN*_0_. All individuals died with *per capita* rate *α*, meaning individuals had a Type II (exponential) survivorship curve. We set the mean life expectancy to be 1/*α* = 75 years. The SEIR model has exact solutions for the basic reproductive number and herd immunity threshold [44], we used these to set the transmissibility of the pathogen *β*(*t*) = *β*_0_, ensuring that *R*_0_ = 10 and the herd immunity threshold was at 90% vaccine coverage.

A summary of the transition rates and effects of the SEIR model are listed in Table 2.

**Table 2:** Transitions of the SEIR process model. At the beginning of each aggregation period the number of new cases, *C*, is reset to 0.

Name	(Δ*S*, Δ*E*, Δ*I*, Δ*R*, Δ*C*)	Propensity
unvaccinated birth	(1, 0, 0, 0, 0)	*α*{1 − *v*(*t*)}*N*_0_
vaccinated birth	(0, 0, 0, 1, 0)	*αv*(*t*)*N*_0_
death of *S*	(−1, 0, 0, 0, 0)	*αS*
death of *E*	(0, −1, 0, 0, 0)	*αE*
death of *I*	(0, 0, −1, 0, 0)	*αI*
death of *R*	(0, 0, 0, −1, 0)	*αR*
importation	(−1, 1, 0, 0, 0)	*ζS*/*N*_0_
transmission	(−1, 1, 0, 0, 0)	*β*(*t*)*SI*/*N*_0_
becoming infectious	(0, −1, 1, 0, 0)	*ρI*
recovery	(0, 0, −1, 1, 1)	*γI*

#### Seasonal SEIR model

The seasonal SEIR model is identical in all respects to the nonseasonal SEIR model, apart from seasonality in the transmission term, with *β* varying over the course of a year dependent on whether schools were open or closed. Using the dates for term times in England listed in [48], the transmission rate was *β*(*t*) = *β*_0_ − *b*_1_ on days when schools were shut and *β*(*t*) = *β*_0_ + *b*_1_*l*/(1 − *l*) when schools were open. The amplitude of seasonality was *b*_1_ = 0.3 (appropriate for measles [44]). The parameter *l* = 0.26 is a normalization constant, and is equal to the fraction of days schools were shut.

#### Age-structured SEIR model

The Age-structured SEIR model used contact rate data from the POLYMOD study [49] to model disease transmission in a population with age-assortative mixing. The model included effects of demographic stochasticity, and was implemented as a discrete time Euler-multinomial process [48]. The simulation time step was set to one day.

The survivorship curve was assumed to be a step function (Type I), with all mortality occurring at age 75 years. The birth rate was fixed to give a constant population size of *N*_0_ = 10^6^ individuals, meaning all ages classes *i* = 1, …, 75 consisted of *N*_*i*_ = *N*_0_/75 individuals.

The force of infection experienced by a susceptible individual in age class *i* was

$$\begin{array}{c} \lambda_{i}=\sum_{j}\beta_{ij}\left(t\right)K_{ij}\frac{I_{j}}{N_{j}}+\frac{\zeta }{N_{0}}, \end{array}$$ (1)

where *ζ*/*N*_0_ is the *per capita* importation rate, *β*_*ij*_(*t*) is the transmission probability, *K*_*ij*_ is the rate an individual of age class *i* contacts individuals in age class *j* and *I*_*j*_ is the number of infectious individuals in age class *j*. If either *i* or *j* were of school age (5-15 years old) then the transmission rate *β*_*ij*_(*t*) was subject to the same term time forcing as the seasonal SEIR model, otherwise it is constant *β*_*ij*_ = *β*_0_. The transmission coefficient *β*_0_ was set to give an *R*_0_ = 10 (calculated using the next-generation matrix [50]), matching the SEIR model.

The contact matrix *K*_*ij*_ was derived from the POLYMOD matrix for Great Britain (Table S8.3 of [49]) via two steps. First, the POLYMOD matrix, with elements *Q*_*a*,*b*_, was symmetrised to correct for reciprocity via [48]

$$\begin{array}{c} {\bar{Q}}_{a,b}=\left(N_{a}Q_{a,b}+N_{b}Q_{b,a}\right)/2N_{a}, \end{array}$$ (2)

where *a* and *b* label the age categories of the POLYMOD matrix (14 5-year increments ranging from 0–70 and 70+). Second, the contact matrix *K*_*i*,*j*_ was constructed from

$$\begin{array}{c} K_{i,j}={\bar{Q}}_{a_{i},b_{j}}N_{j}/N_{b_{j}}, \end{array}$$ (3)

where *a*_*i*_ and *b*_*j*_ label the age categories of the POLYMOD matrix that *i* and *j* respectively belong to. Given the flat population profile from ages 0 to 75, $N_{a_{i}}=5/75$ and $N_{i}/N_{a_{i}}=1/5$ for all *i* = 0…, 75.

#### FRED model

FRED is an open-source agent-based simulator that simulates disease transmission in synthetic populations [35]. The simulator is designed to capture the spatial and demographic heterogeneities of a specific population by constructing a synthetic population matched to census data for a given geographic region [51]. We used the pre-constructed synthetic population for Allegheny county (Pittsburgh), Pennsylvania, USA [35].

FRED explicitly represents each individual in the population as an agent, who each have a record of demographic traits (e.g. age, employment status, family income), health status (e.g. vaccine status, infectivity) and locations of social activity (e.g. household, school, workplace). FRED implements demographic dynamics, with individuals born, aging, and dying according to the synthetic population’s birth rates and age-specific mortality rates [35]. Infection status follows the SEIR pattern, as used in the other models studied in this paper. At each time step (fixed to one day) infectious agents visit the various locations of social activity and can transmit the infection to other agents also present. Transmission is only possible between agents present at the same location, and occurs with a probability dependent on the ages of the two agents. Transmission is seasonal, with schools closed during the summer holidays and on weekends, and most workers do not attend workplaces at the weekend. The transmissibility of the pathogen was tuned to ensure a similar herd immunity threshold to the SEIR model.

A complete description of the simulator is beyond the scope of this paper, we refer the reader to [35] and the FRED documentation, available online at https://fred.publichealth.pitt.edu. All FRED configuration parameters necessary to reproduce the results of this paper are listed in S1 Table.

#### Mplex model

The Mplex model [37] simulated disease transmission on a multiplex network consisting of three layers (the household, school, and community layers), following the SEIR scheme adopted by the other models presented in this work. The multiplex network comprises of about 10^6^ nodes and was constructed using Italian socio-demographic data [52]. A brief description of the model is presented here, with a full description provided in S1 Text.

Each individual in the population was represented by a unique node in the network. Individuals were assigned an age, resolved in years and days. At each simulation time step (corresponding to 1 day), three demographic events were simulated [53]: i) individuals could die with a probability given by the age-specific daily mortality rate of the Italian population; ii) for each deceased individual a newborn individual was introduced to the population, guaranteeing that the population size remains constant and at a demographic equilibrium; iii) the age of all (alive) individuals was increased by 1 day. Once per year, school-age individuals were reassigned to a school appropriate for their age. In addition to the demographic process, at each time step of the simulation the Mplex model simulated disease transmission dynamics. During regular school days, the transmission can occur in each of the three layers, while during the summer holidays no transmission at school is possible. Layer-specific weights regulating the transmission process in each layer were estimated from the Italian time-use data by assuming that the transmission probability is proportional to the time spent in contact with other individuals [54]. The latent period, the infectious period, and the case importation rate were the same as for the other models. The transmission rate was set to obtain *R*_0_ = 10.

### Estimating the time of emergence

To establish whether CSD was present prior the epidemic transition, we needed to determine the timing of the epidemic transition, i.e. the time at which *R*_eff_ = 1. For the nonseasonal SEIR model, an analytical expression exists for *R*_eff_(*t*) allowing the time of emergence to be found algebraically. For higher-dimensional models with seasonality we found the time of emergence by fitting a Poisson transmission model using Bayesian MCMC.

#### Poisson transmission model

The Poisson transmission model is a one-dimensional non-Markovian Poisson process that models the number of new cases through time, based on the renewal equation [55]. Versions of this model have been used to model the transmission of Ebola [36, 56] and Influenza [37].

The model assumes that the number of new cases at time step *t* + *δ*, denoted *C*_*t*+*δ*_, follows a Poisson distribution *C*_*t*+*δ*_ ∼ Poisson(λ_*t*_) with rate parameter

$$\begin{array}{c} \lambda_{t}=\delta (R_{\text{eff}}\left(t\right)\sum_{s\leq t}\phi \left(t-s\right)C_{s}+\eta ), \end{array}$$ (4)

where *R*_eff_(*t*) is the effective reproductive number, *ϕ*(*t* − *s*) is the infectiousness kernel and *η* is the rate cases are imported. The infectiousness kernel *ϕ*(*t* − *s*) is given by

$$\begin{array}{c} \phi \left(t-s\right)=\frac{\int_{t-s}^{t-s+\delta }dt^{\prime }\chi \left(t^{\prime }\right)}{\int_{0}^{\infty }dt^{\prime }\chi \left(t^{\prime }\right)}, \end{array}$$ (5)

where *χ*(*t*′) is the probability that an individual is infectious *t*′ after infection. We assumed exponentially distributed latent and infectious periods, giving

$$\begin{array}{c} \chi \left(t^{\prime }\right)=\frac{\rho }{\rho -\gamma }[e^{-\gamma t^{\prime }}-e^{-\rho t^{\prime }}], \end{array}$$ (6)

where *ρ* and *γ* are the rates of the latent and infectious period distributions, respectively.

Cases stemming from external importation occur with rate weighted by the fraction of the population susceptible, initially *η* = (1 − *v*(0))*ζ*. As vaccination decreases the importation rate will rise, however this increase is much less relative to the increase in secondary transmission. To reduce the number of parameters estimated by MCMC, we therefore fixed the importation rate at the initial value.

The effective reproductive number was modeled using the piecewise function

$$\begin{array}{c} R_{\text{eff}}\left(t\right)=\{\begin{array}{cc} R_{\text{eff}}^{i} & \text{if}\;t<0,\\ R_{\text{eff}}^{i}+\left(1-R_{\text{eff}}^{i}\right)(\frac{t}{\Delta }{)}^{\epsilon } & \text{if}\;0\leq t<\Delta ,\\ 1 & \text{if}\;\geq \Delta , \end{array} \end{array}$$ (7)

where vaccine uptake starts to decreased at time *t* = 0. The parameter *ϵ* controls the curvature of *R*_eff_(*t*). The analytical solution for susceptible replenishment in the nonseasonal SEIR model with declining vaccine uptake can be well-approximated by a quadratic function, therefore we set *ϵ* = 2. The two model parameters which required estimation were the time of emergence, Δ, and the initial reproductive number $R_{\text{eff}}^{i}$.

#### Bayesian Markov Chain Monte Carlo

The two unknown parameters ($R_{\text{eff}}^{i}$ and Δ) were estimated by sequentially fitting the Poisson transmission model to each simulated realisation using Bayesian MCMC. Each time series was of weekly case reports (i.e. *δ* = 1 week) between *t*_0_ = −10 years and *T* = 40 years. Using the Poisson transmission model, the probability of observing a time series $C={\left\{C_{t}\right\}}_{t=t_{0}}^{T}$ is

$$\begin{array}{c} P\left(C|\Theta \right)=\prod_{t=t_{0}}^{T-\delta }P\left(C_{t+\delta }|{\left\{C_{s}\right\}}_{s=t_{0}}^{t}\right), \end{array}$$ (8)

where $P(C_{t+\delta }|{\left\{C_{s}\right\}}_{s=t_{0}}^{t})$ is a Poisson distribution with rate parameter given in Eq 4 and $\Theta =\{\Delta ,R_{\text{eff}}^{i}\}$. We assumed that before *t* = *t*_0_ there are no cases, for *t*_0_ ≪ 0 this has negligible effect on parameter estimates.

By applying Bayes’ rule iteratively, the joint posterior density for the parameters, given the first *i* simulated time series, is

$$\begin{array}{c} \pi^{i}\left(\Theta |{\left\{C_{j}\right\}}_{j=1}^{i}\right)\propto P\left(C_{i}|\Theta \right)q^{i}\left(\Theta \right), \end{array}$$ (9)

where ***C***_*i*_ = {*C*_*i*,*t*_}_*t*_ is the *i*-th time series of cases and *P*(***C***_*i*_|Θ) is given in Eq 8. For *i* ≥ 2, the prior is equal to the preceding posterior $q^{i}=\pi^{i-1}\left(\Theta |{\left\{C_{j}\right\}}_{j=1}^{i-1}\right)$. We assumed the initial prior, *q*^1^, was uniform for Δ ∈ (0, *T*] years and $R_{\text{eff}}^{i}\in \left[0,1\right)$.

We generated 30000 samples from the posterior by running Hamiltonian Monte Carlo with the No-U-Turn Sampler [57] implemented in the python package *pymc3* [58]. We then constructed a smoothed posterior distribution from the samples using Gaussian kernel density estimation [59]. This smoothed posterior was then fed back into the MCMC algorithm as the subsequent prior, and the procedure was repeated.

We obtained point estimates $\hat{\Theta }=\left\{\hat{\Delta },{\hat{R}}_{\text{eff}}^{i}\right\}$ from the *maximum a posteriori* of the final posterior given all *M* = 100 time series,

$$\begin{array}{c} \hat{\Theta }=\text{arg}\;\underset{\Theta }{\text{max}}\;\pi^{M}\left(\Theta |{\left\{C_{j}\right\}}_{j=1}^{M}\right). \end{array}$$ (10)

### Critical slowing down and early-warning signals

#### Critical slowing down

In a previous theoretical study using the Birth-Death-Immigration (BDI) process, a simple transmission model that ignores any effects of susceptible depletion, the presence of CSD was shown using the autocorrelation [7]. For a subcritical (*R*_eff_ < 1) disease, the BDI process can be solved to give an expression for the autocorrelation in the number of individuals infected at lag *δ* [2, 7],

$$\begin{array}{c} \text{AC}\left(\delta \right)=e^{-(1-R_{\text{eff}})\gamma \delta }. \end{array}$$ (11)

As *R*_eff_ increases the autocorrelation also increases, approaching one as *R*_eff_ → 1. The increase in the autocorrelation is caused by the increasing persistence of perturbations that defines CSD [6, 7]. In line with this theoretical result, we took an increasing trend in the autocorrelation prior to the epidemic transition as evidence for the presence of CSD. Using 100 simulated time series, we numerically calculated the autocorrelation at lag one month through time for each model.

#### Estimating EWS

A range of EWS have been proposed to anticipate dynamical transitions [5–7, 10, 28, 30]. We considered seven: the autocorrelation (at lag 1 month), coefficient of variation, index of dispersion, kurtosis, mean, skewness and variance. EWS were calculated for each simulated time series of case counts. Prior to calculating the EWS we grouped the weekly counts into 4-weekly counts, as a previous study into EWS using imperfect data found that this resulted in more robust performance [28].

Each EWS was calculated longitudinally from a single realization using a moving window estimator [6, 7]. We chose to use exponentially weighted moving averages; for example the estimator for the mean is

$${\hat{\mu }}_{i,t}=Z^{-1}\sum_{s=t_{0}}^{t}e^{-\kappa (t-s)}C_{i,s},$$ (12)

$$Z=\sum_{s=t_{0}}^{t}e^{-\kappa (t-s)},$$ (13)

and for the variance is

$${\hat{\sigma }}_{i,t}^{2}=Z^{-1}\sum_{s=t_{0}}^{t}e^{-\kappa (t-s)}{\left(C_{i,s}-{\hat{\mu }}_{i,s}\right)}^{2}.$$ (14)

The decay rate is specified by the half-life *t*_1/2_ = ln(2)/*κ*. We set *t*_1/2_ = 39 4-week intervals, which is approximately 3 years. The estimators for the remaining EWS were constructed similarly, and are shown in Table 3.

**Table 3:** List of early-warning signals and estimators.

EWS	Estimator
Mean	${\hat{\mu }}_{t}=\sum_{s=t_{0}}^{t}\frac{e^{-\kappa (t-s)}C_{s}}{Z}$
Variance	${\hat{\sigma }}_{t}^{2}=\sum_{s=t_{0}}^{t}\;\;\frac{e^{-\kappa (t-s)}{\left(C_{s}-{\hat{\mu }}_{s}\right)}^{2}}{Z}$
Coefficient of variation	${\hat{\text{CoV}}}_{t}=\frac{{\hat{\sigma }}_{t}}{{\hat{\mu }}_{t}}$
Index of dispersion	${\hat{\text{IoD}}}_{t}=\frac{{\hat{\sigma }}_{t}^{2}}{{\hat{\mu }}_{t}}$
Skewness	${\hat{\text{Skew}}}_{t}=\frac{1}{{\hat{\sigma }}_{t}^{3}}\sum_{s=t_{0}}^{t}\;\;\frac{e^{-\kappa (t-s)}{\left(C_{s}-{\hat{\mu }}_{s}\right)}^{3}}{Z}$
Kurtosis	${\hat{\text{Kurt}}}_{t}=\frac{1}{{\hat{\sigma }}_{t}^{4}}\sum_{s=t_{0}}^{t}\;\;\frac{e^{-\kappa (t-s)}{\left(C_{s}-{\hat{\mu }}_{s}\right)}^{4}}{Z}$
Autocorrelation at lag *δ*	${\hat{\text{AC}}}_{t}=\frac{1}{{\hat{\sigma }}_{t}{\hat{\sigma }}_{t-\delta }}\sum_{s=t_{0}}^{t}\;\;\frac{e^{-\kappa (t-s)}\left(C_{s}-{\hat{\mu }}_{s}\right)\left(C_{s-\delta }-{\hat{\mu }}_{s-\delta }\right)}{Z}$

### Quantifying performance using the AUC statistic

Following a previous study [28], we scored performance using the Area Under the ROC Curve (AUC) statistic, which quantifies how successfully a particular EWS classifies whether or not a disease is approaching an epidemic transition [60].

The Reciever Operator Characteristics (ROC) curve is a parametric plot of the sensitivity and specificity of a classification method as a function of the detection threshold [60]. As null (not emerging) data we took all EWS values in the interval −5 < *t* < 0 years, i.e. immediately before vaccine uptake started dropping and the pathogen started re-emerging. The test data were then the EWS values for *t* > 0 years. We calculated the ROC and AUC using data for each time point separately, to show how the detectability of emergence changes with time.

The AUC statistic quantifies the overlap of test and null distributions, and may be interpreted as the probability that the EWS at time *t* from a randomly chosen realisation is higher than a randomly chosen value from the null interval −5 < *t* < 0 years, AUC = *P*(*τ*_test_ > *τ*_null_) [60].

An AUC = 0.5 implies that an observed EWS value conveys no information about whether or not the disease is re-emerging. An AUC greater than (less than) 0.5 implies that test values are typically larger (smaller) than null values. Given AUC values further from 0.5 imply better performance, and some EWS may increase or decrease as the transition is approached, we compared performance using the absolute distance |AUC − 0.5|. Performance is maximised if |AUC − 0.5| = 0.5. We also calculated the sign of (AUC − 0.5), to see whether an EWS consistently increased/decreased for all models and times.

## Supporting information

S1 Text Description of the Mplex model. (PDF)

S1 Fig Area under the ROC curve (AUC) through time. a–g) AUC through time for each model for the EWS indicated in the panel. Vertical lines indicate the estimated time of emergence.
(TIFF)

S1 Table List of model parameters for FRED. (PDF)
