# Supplementary material for: Detecting critical slowing down in high-dimensional epidemiological systems
Source: PLoS Comput Biol. 2020 Mar 9;16(3):e1007679. doi: 10.1371/journal.pcbi.1007679 (PMC7082051; doi:10.1371/journal.pcbi.1007679)
Supplement: S1 Text — (PDF) [file pcbi.1007679.s001.pdf]

Detecting critical slowing down in high-dimensional epidemiological dynamics

Tobias Brett[1,2], Marco Ajelli[3,4], Quan-Hui Liu[3,5], Mary G. Krauland[6], John J. Grefenstette[6], Wilbert G. van Panhuis[7,8], Alessandro Vespignani[3,9], John M. Drake[1,2], Pejman Rohani[1,2,10]

[1] Odum School of Ecology, University of Georgia, Athens, GA, USA
[2] Center for the Ecology of Infectious Diseases, University of Georgia, Athens, GA, USA
[3] Laboratory for the Modeling of Biological and Socio-technical Systems, Northeastern University, Boston, MA, USA
[4] Bruno Kessler Foundation, Trento, Italy
[5] College of Computer Science, Sichuan University, Chengdu, China
[6] Department of Health Policy and Management, University of Pittsburgh, Pittsburgh, PA, USA
[7] Department of Epidemiology, University of Pittsburgh, Pittsburgh, PA, USA
[8] Department of Biomedical Informatics, University of Pittsburgh, Pittsburgh, PA, USA
[9] ISI Foundation, Turin, Italy
[10] Department of Infectious Diseases, College of Veterinary Medicine, University of Georgia, Athens, GA, USA

S1 Text

1 Description of the Mplex model

The Mplex model simulates the demographic evolution of a population and measles transmission dynamics on a time-varying multiplex network consisting of three layers (the household, school, and community layers), where each node in the network represents an individual. In this appendix we provide a detailed description of all the ingredients of the model.

1.1 Demographic model

To set the initial condition of the demographic model, we followed the algorithm introduced by Fumanelli et al [1] to generate a subsample of the Italian population consisting of about 1,000,000 individuals catagorised by age (expressed in years and days) and grouped into households and schools (school types considered: kindergarten, primary school, middle school, and high school). School sizes were appropriate according to age. The socio-demographic data used to define this initial population were taken from public records available at `demo.istat.it` and `https://ec.europa.eu/eurostat/data/database`.

To simulate the evolution of the population over time, we considered the following demographic processes:

- **Death process.** At each time step of the simulation ($\Delta t = 1$ day) and for each individual i, we determined whether an individual survives or died by sampling a random value from a Bernoulli distribution of probability $m(a_i)$, where a_i is the age of individual i expressed in years and $m(a)$ is the daily mortality rate for individuals aged a years. We defined $m(a)$ according to the yearly mortality rates of the Italian population (see Fig. S1A). Note that we arbitrarily set $m(100) = 1$, which corresponds to an upper limit of 100 years on the age of the individuals in the model.

- **Birth process.** At each time step of the simulation, we set the number of births to be equal to the number of death occurring at that time step. This ensured a constant total number of individuals in the population. This procedure corresponds to an average annual birth rate of the population of 0.013,

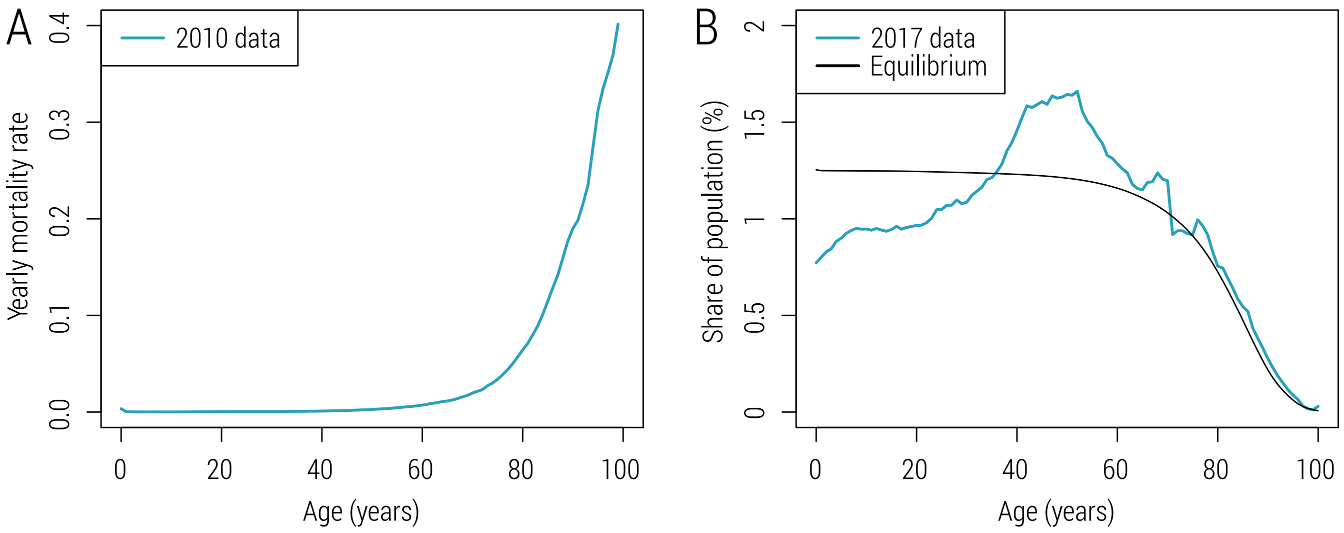

Figure S1: **A** Yearly mortality rates by age for the Italian male population as of 2010. Note that to obtain the daily mortality rates by age used in the model it is necessary to divide the values reported in the figure by 365. **B** Age structure of the equilibrium population simulated by the model and, for comparison, the 2017 age structure of the Italian population that was used as initial condition.

comparable with other models. The obtained age-structure of the equilibrium population is shown in Fig. S1B. Finally, newborns were assigned to households ensuring that the share of susceptible individuals by household size is preserved and their age was set to 0 years and 1 day.

- **Aging process.** At each time step of the simulation, the age of all individuals was increased by one day.

In addition to these three demographic processes that occurred at each time step of the simulation (i.e., daily), once per simulated year individuals are reassigned to a school appropriate for their age. This update was performed at the time step corresponding to September 14 of each simulated year, the last day of summer vacation in our model (see Sec. 1.2). To determine whether an individual was a student or a teacher, we use Italian occupation rates by age (see Fig. S2 – data available at `https://ec.europa.eu/eurostat/data/database`).

We would like to stress that the demographic equilibrium depends on the birth and age-specific mortality rates, while the initial condition essentially determines the number of time steps required to reach the equilibrium. As such, the most important parameters specific to the Italian population that determine the demographic equilibrium and the composition of the schools are, respectively, the mortality rates by age (Fig. S1A) and the occupation rates by age (Fig. S2).

Multiplex network. The procedure we have described thus far defines a synthetic population in which an individual is characterized by an age and belongs to a household and (potentially) to a school. This structure allowed us to define a multiplex network consisting of three layers (i.e., the household, school, and community layers) where each individual corresponds to node in each layer [2]. The household layer was composed by N_h disconnected clusters, each representing a unique household. The school layer is composed by N_s disconnected clusters, each representing a unique school. Note that, formally, an individual who does not attend school (for instance because she is beyond the school age) is represented in the school layer as a node with no links. The resulting degree distributions of the household-layer and school-layer networks are shown in Fig. S3. Finally, the community layer is made of a fully connected layer corresponding to a perfectly random mixing model.

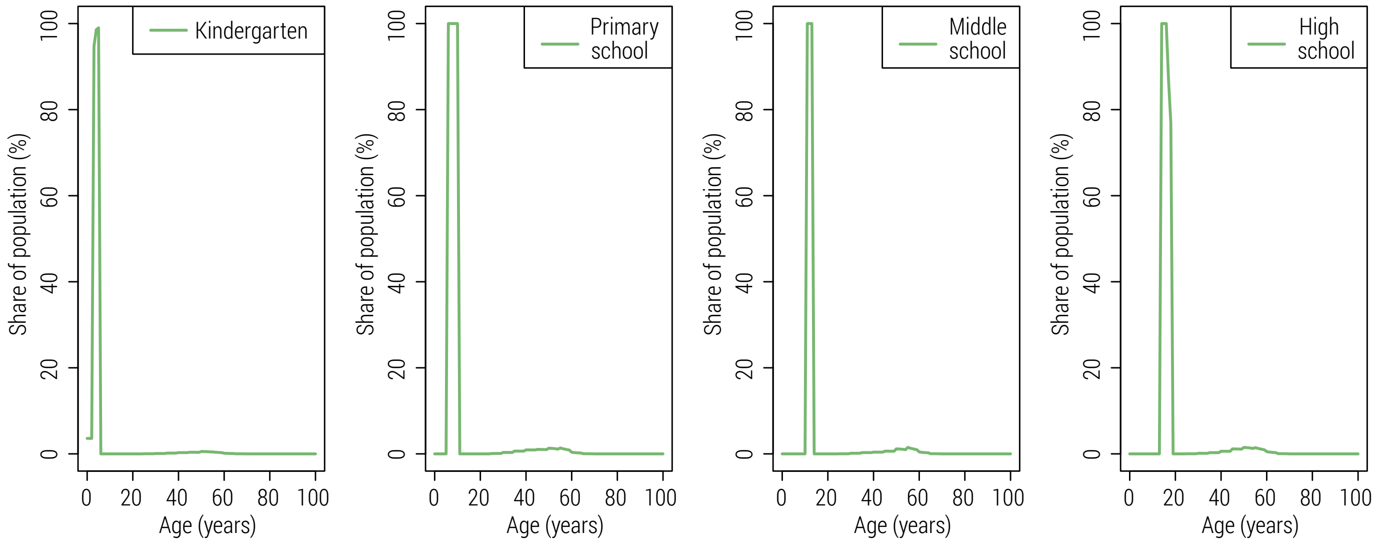

Figure S2: Share of population attending educational institutions (either as student or teacher) by age and school type.

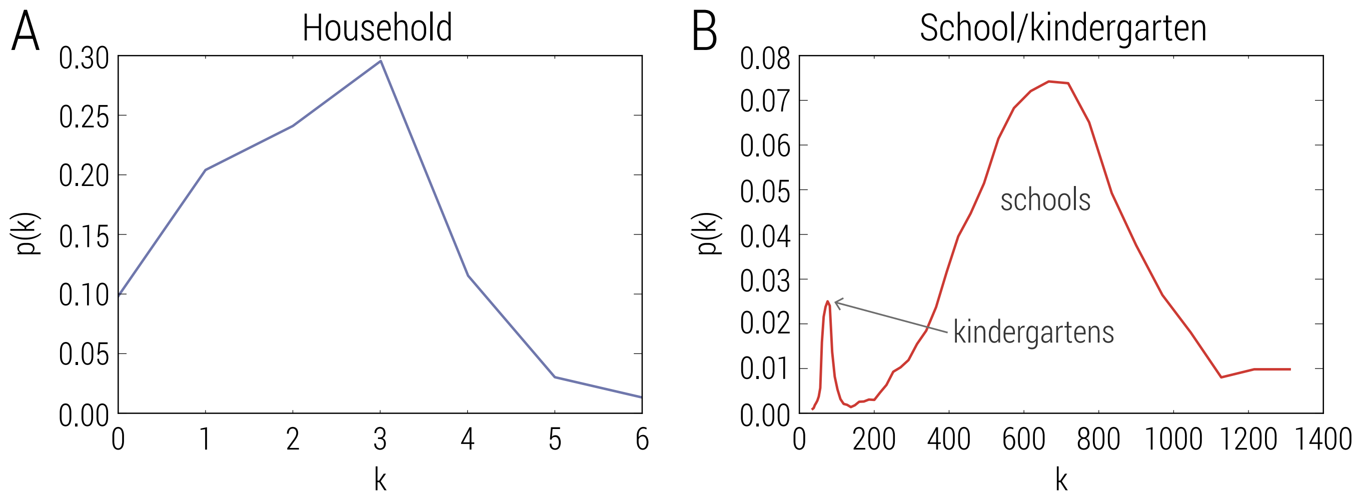

Figure S3: Degree distributions of the household-layer (**A**) and school-layer (**B**) networks.

1.2 Transmission model

We model measles transmission following the Susceptible-Exposed-Infectious-Removed SEIR scheme. The removed class is sub-divided into individuals removed due to: i) recovery from infection ii) vaccination (see Fig. S4A). Each node of the multiplex network occupies one of these epidemiological statuses (i.e., susceptible, exposed, infectious, removed-recovered, and removed-vaccinated) at each time step of the simulation. We would like to stress that the node has the same epidemiological status in all layers; thus, as soon as a node changes its status in one layer, the status is instantly updated in all layers. The structure of the Mplex model is shown in Fig. S4B.

The disease transmission dynamics in the Mplex model were implemented according to the following scheme. At each time step of the simulation:

- **Transmission of infection.** The probability that infectious individual i transmitted the infection to susceptible individual j was

$$p_{i \to j} = \beta \sum_{\alpha} a_{\alpha}^{i,j} w_{\alpha}/(n_{\alpha} - 1), \tag{1}$$

  where

  - $\alpha = \{\text{household, school, community}\}$ indicates the layer;
  - $a_{\alpha}^{i,j}$ is the adjacency matrix on layer α (i.e., $a_{\alpha}^{i,j} = 1$ if individuals i and j are connected on layer α, 0 otherwise);
  - n_{α} is the average cluster size in layer α (i.e., the average household size for the household layer, the average school size for the school layer, and the total number of individuals in the population for the community layer);
  - w_{α} weights the transmission on layer α;
  - β is the transmission rate.

- **Transition from exposed to infectious.** At every time step an individual is in the exposed state, we determined whether she enters the infectious compartment by sampling a random value from a Bernoulli

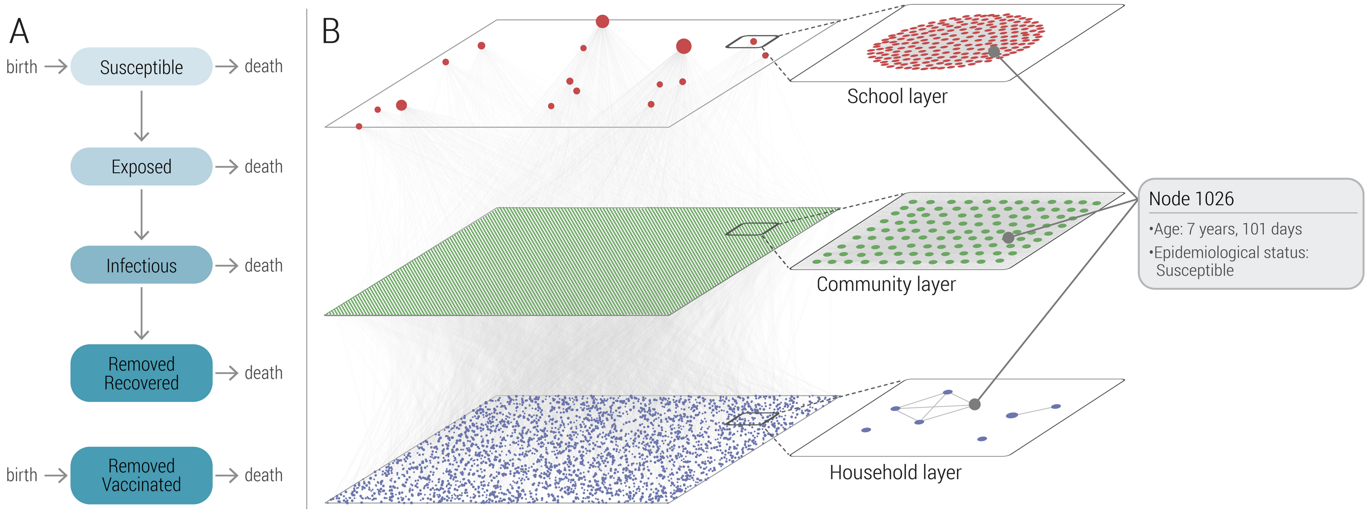

Figure S4: **A** Epidemic flow of the SIER model with birth and death process. **B** Graphical representation of the structure of the Mplex model. The figure refers to a subsample of 10,000 nodes at time step 1. The birth and death processes as well as the grouping of new students in schools at the beginning of each school year allow the structure of the network to evolve over time. Note that the community layer is a complete graph, although not all edges are visible for the sake of readability of the illustration. Moreover, in each layer are shown only nodes with at least one link.

distribution of probability $1/8$ days^{-1}. The resulting distribution of the latent periods is an exponential distribution with mean 8 days.

- **Recovery from the infection.** At every time step an individual in the infectious state, we determined whether she enters the removed-recovered compartment by sampling a random value from a Bernoulli distribution of probability $1/5$ days^{-1}. Again, the resulting distribution of the infectious periods is an exponential distribution with mean 5 days.
- **Vaccination.** In all models presented in this study, we assumed that individuals receive a perfect vaccine (i.e., with no primary vaccine failure, leakiness or waning of immunity) at birth. Therefore, in the Mplex model, when a new individual is born we determined whether she is susceptible or removed-vaccinated by sampling a random value from a Bernoulli distribution of probability equal to the vaccination uptake

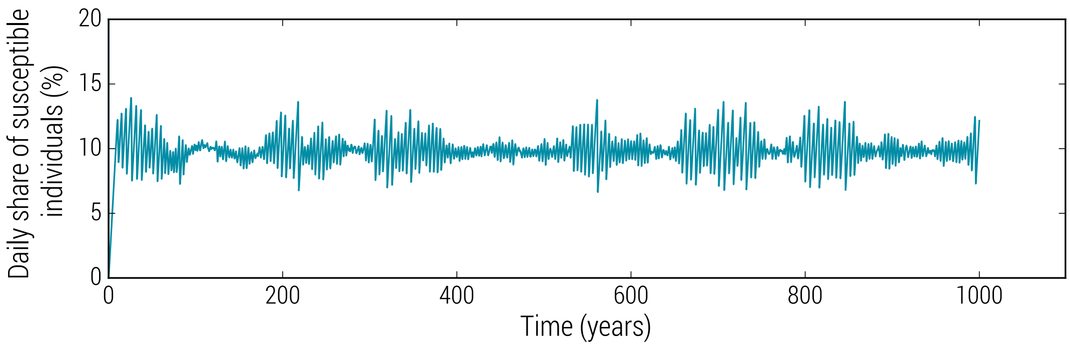

Figure S5: Daily share of susceptible individuals in the population in a randomly selected simulation of the Mplex model obtained by considering a constant vaccine uptake of 0%.

in that calendar year.

- **Importation of cases.** To simulate the importation of cases from outside sources we used a procedure also adopted by other models presented in this study. We uniformly sample one node of the network every 7 time steps (i.e., once per simulated week) and if the sampled node is in the susceptible status we change its status to exposed. In a fully susceptible population, this procedure corresponds to one imported case per week.

Calibration of the transmission model. We assumed that the transmission probability given a contact in a specific social setting (e.g., the school) is proportional to the time spent in contact by the infectious and susceptible individuals [3]. Therefore, the layer-specific weights w_α in Eq. (1) were inferred from the Italian time-use data (which is available at `http://dati.istat.it/Index.aspx`). The resulting weights are: $w_{\text{household}} = 0.746$, $w_{\text{community}} = 0.288$, and $w_{\text{school}} = 0.200$. We consider a 3-month summer vacation according to the Italian school calendar, during which no transmission occurred via contacts on the school layer (i.e. $w_{\text{school}} = 0$). The summer vacation was set to start on June 15 and to end on September 14 of each simulated year. No other holidays were considered throughout the year.

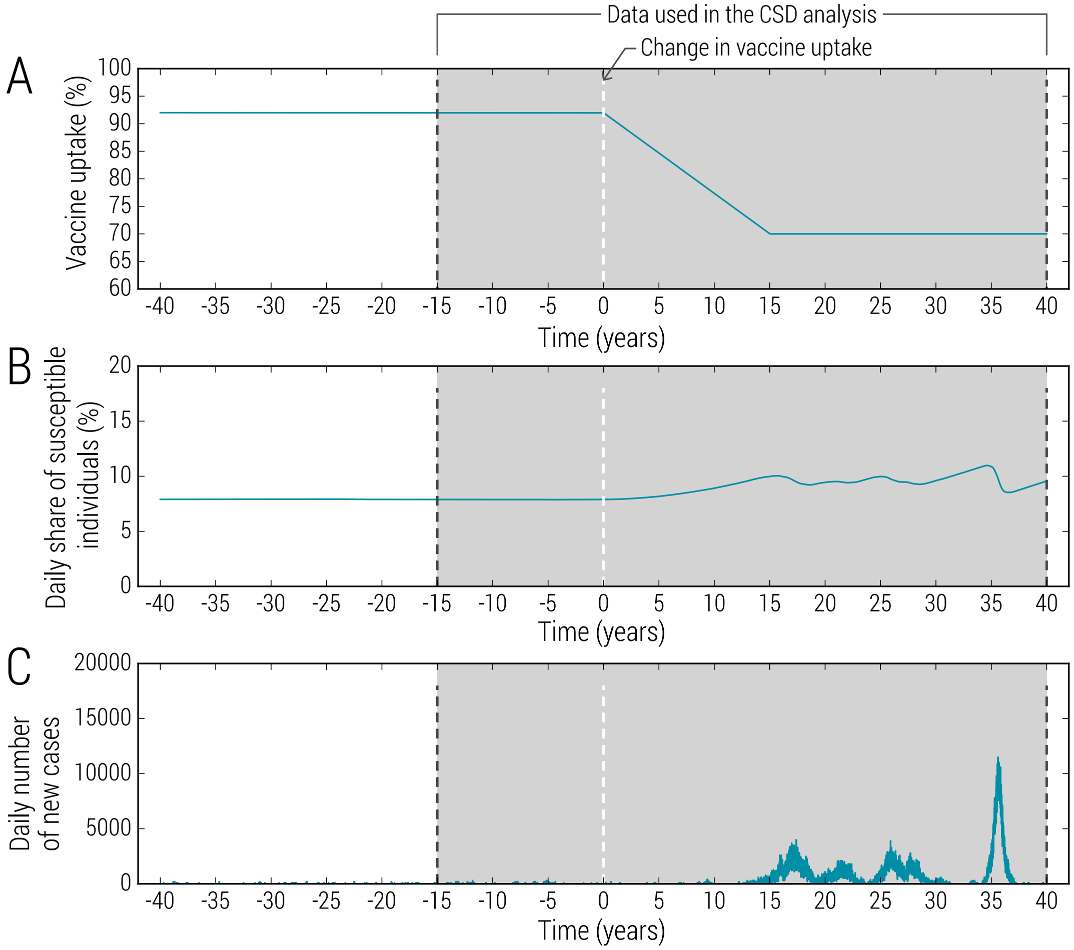

Figure S6: **A** Vaccine uptake over time used in the analysis of CSD. **B** Daily share of susceptible individuals in the population in a randomly selected simulation of the Mplex model. **C** Daily number of new infectious individuals in the same simulation shown in panel B.

After setting the layer-specific weights, and given that all the other model parameters (e.g., duration of the latent period, recovery rate) were fixed, the infection transmission process was regulated by one single parameter: the transmission rate, β. Therefore, by setting β we univocally determine the value of R_0. Given the complexity of the Mplex model, we do not have an explicit equation to calculate R_0. We thus followed the arguments of Anderson and May [4] to define

$$R_0 = 1/S^\star$$

where $S^\star$ is the share of susceptible individuals at the endemic equilibrium.

As in the other models compared in this study, we wanted $R_0 = 10$ (i.e., $S^\star = 0.1$). We explored a range of values of β, running the model until it reaches both the demographic and endemic equilibrium assuming vaccine uptake fixed at 0, and calculated $S^\star$ (see Fig S5). By using this procedure, we estimated $\beta = 0.6$.

To perform the simulation used in the analysis of the CSD, we initialized the system with a population having 90% of removed individuals, considered a constant vaccine uptake of 92%, and run the Mplex model for 1,000 (simulated) years to assure that both the demographic dynamics and transmission dynamics were at equilibrium. We then considered the vaccine uptake of new borns to drop linearly from 92% to 70% over 15 years and then remain constant at 70% for 50 years (see Fig S6A). Finally, we consider in the analysis of CSD using only data in the time interval [-15, +40] years with 0 indicating the time at which vaccine uptake started to drop. Representative simulated time series for the fraction of the population susceptible and daily new cases are shown in Fig S6B and C, respectively.

2 References

[1] Fumanelli L, Ajelli M, Manfredi P, Vespignani A, Merler S (2012) Inferring the structure of social contacts from demographic data in the analysis of infectious diseases spread. *PLoS Comput Biol* 8(9):e1002673.

[2] Liu QH et al. (2018) Measurability of the epidemic reproduction number in data-driven contact networks. *Proc Natl Acad Sci USA* 115(50):12680–12685.

[3] Ajelli M, Poletti P, Melegaro A, Merler S (2014) The role of different social contexts in shaping influenza transmission during the 2009 pandemic. *Sci Rep* 4:7218.

[4] Anderson RM, May RM (1991) *Infectious diseases of humans: dynamics and control.* (Oxford University Press; Oxford, UK).
