# Supplementary figures and images for: Detecting critical slowing down in high-dimensional epidemiological systems
Source: PLoS Comput Biol. 2020 Mar 9;16(3):e1007679. doi: 10.1371/journal.pcbi.1007679 (PMC7082051; doi:10.1371/journal.pcbi.1007679)

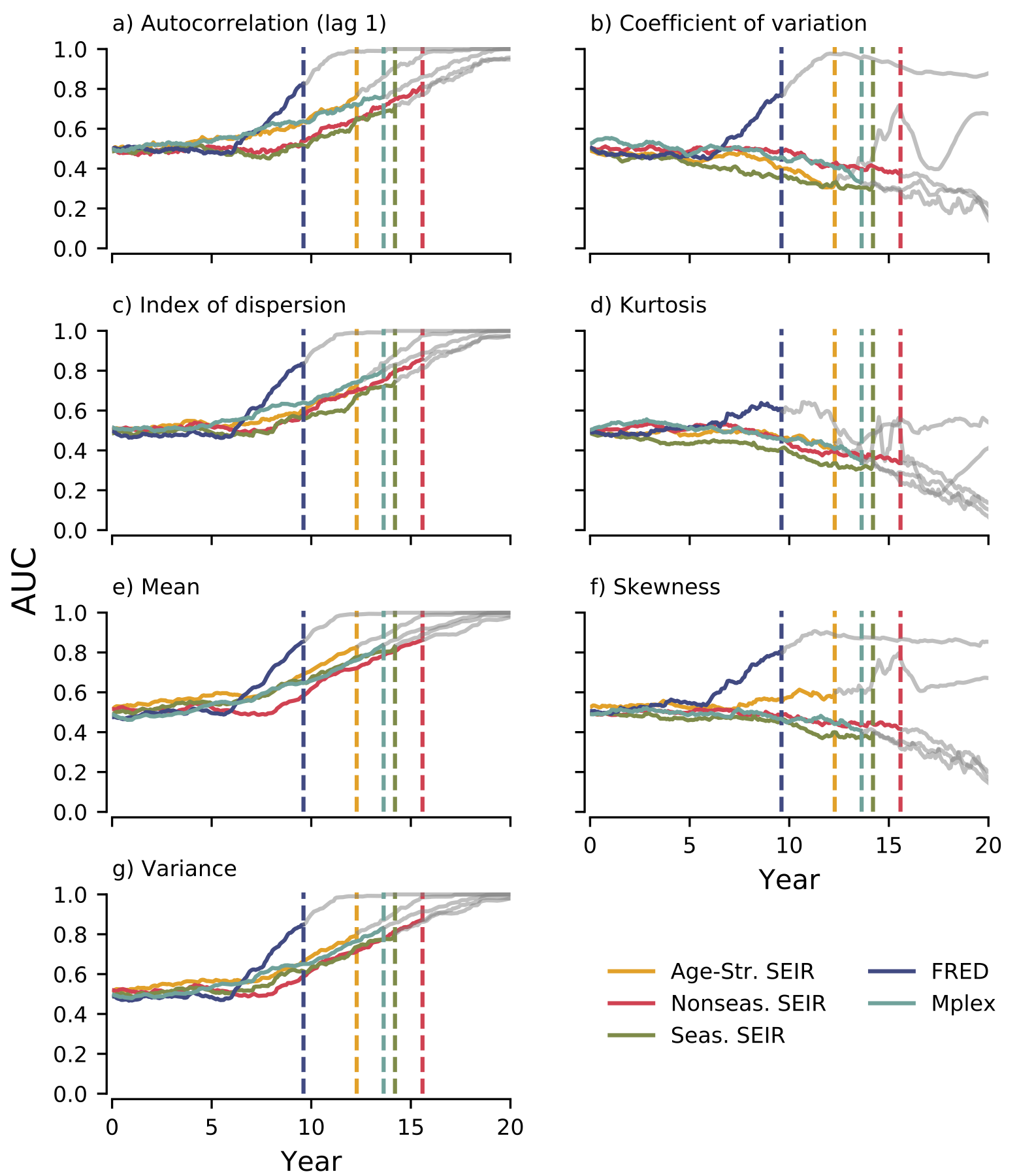

Supplement: S1 Fig — a–g) AUC through time for each model for the EWS indicated in the panel. Vertical lines indicate the estimated time of emergence. (TIFF) [file pcbi.1007679.s002.tiff]
