# Supplementary material for: Detecting critical slowing down in high-dimensional epidemiological systems
Source: PLoS Comput Biol. 2020 Mar 9;16(3):e1007679. doi: 10.1371/journal.pcbi.1007679 (PMC7082051; doi:10.1371/journal.pcbi.1007679)
Supplement: S1 Table — (PDF) [file pcbi.1007679.s003.pdf]

S1 Table: User-modifiable disease-specific parameters

Parameter Type	Parameter	Definition	Value
Natural history parameters	Days latent	Discrete cdf for number of days between becoming exposed and becoming infectious	Lognormal, Median 8.0, Dispersion 1.5
	Symptomatic rate	The probability of an infected person becoming symptomatic	1.0
	Days asymptomatic	Discrete cdf for number of days the agent is infectious and asymptomatic	Not used
	Days symptomatic	Discrete cdf for number of days the agent is infectious and symptomatic	Lognormal, Median 5.0, Dispersion 1.5
	Immunity Loss Rate	Rate at which a person loses immunity after recovering from infection	No loss of immunity
	Mortality Rate	The probability of an infected person dying	No death due to infection
Contact parameters	Probability of staying home	The baseline probability that an agent stays home if the agent experiences a symptomatic infection	No stay-at-home behavior
	Household contact rates	The expected number of potentially infective daily contacts between an infectious agent and a susceptible agent in a household	Household.contacts = 0.3421<br>Household.same_age_bias = 0.05
	Neighborhood contact rate	The expected number of potentially infective daily contacts between an infectious agent and a susceptible agent in a neighborhood	Neighborhood.contacts = 0.7495<br>Neighborhood.same_age_bias = 0.1<br>Neighborhood.weekend_contact_rate = 1.5
	School contact rates	The expected number of potentially infective daily contacts between an infectious agent and a susceptible agent in a school	School.contacts = 0.4159<br>Classroom.contacts = 0.8318
	Workplace contact rates	The expected number of potentially infective daily contacts between an infectious agent and a susceptible agent in a workplace	Workplace.contacts = 0.0679<br>Office.contacts = 0.1358
Transmission parameters	Transmissibility	The transmissibility of disease relative to an arbitrary baseline set by calibration	2.0
	Asymptomatic infectivity	Multiplier for how infective an asymptomatic infected agent is, relative to an symptomatic agent	Not used

FRED includes natural history and transmission parameters for pandemic influenza as used in previous models. Contact parameters were calibrated for the FRED synthetic population using the methods described in [1]. All contact rates are positive real numbers. For more details about these and other user-settable parameters, please see the FRED User Guide.

References

1. Cooley P, Brown S, Cajka J, Chasteen B, Ganapathi L, Grefenstette J, et al. The role of subway travel in an influenza epidemic: a New York City simulation. Journal of Urban Health. 2011;88(5):982.
